# Supplementary material for: The extent to which cancer patients trust in cancer-related online information: a systematic review
Source: PeerJ. 2019 Sep 30;7:e7634. doi: 10.7717/peerj.7634 (PMC6776066; doi:10.7717/peerj.7634)
Supplement: Table S2 [file peerj-07-7634-s002.docx]

**Supplemental Table S2:**

**Entire search string for the database Medline.**

| Search Set | Search Terms | Limits |
| --- | --- | --- |
| #1 cancer | neoplasm [ALL] OR neoplasm [ALL] * OR tumor [ALL] OR tumor* [ALL] OR tumour OR tumour* [ALL] OR neoplasia [ALL] OR cancer [ALL] OR cancer* [ALL] OR benign neoplasm [ALL] OR benign neoplasm [ALL] OR carcinoma [ALL] OR carcinoma* [ALL] | None |
| #2 internet/ online/ web | web [ALL] OR web* [ALL] OR online OR online* [ALL] OR internet OR internet* [ALL] OR "world wide web" [ALL] OR web based [ALL] OR web-based [ALL] OR net [ALL] OR cyberspace [ALL] OR interweb [ALL] OR www [ALL] OR cyber* [ALL] | None |
| #3 health information/ information in general | information* [ALL] OR “Health Information” [ALL] OR info* [ALL] OR advice [ALL] OR data [ALL] OR messag* [ALL] OR news [ALL] OR notification* [ALL] OR fact* [ALL] OR material [ALL] OR clue[ALL] | None |
| #4 trust | trust* [ALL] OR faith* [ALL] OR *entrustment* [ALL] OR credibil* [ALL] OR confidence [ALL] OR plausib* [ALL] OR belief [ALL] OR believing [ALL] OR rely [ALL] OR reliability [ALL] OR integrity [ALL] OR credibleness [ALL] OR accept* [ALL] OR accuracy* [ALL] OR accurate* [ALL] | None |
