# Supplementary material for: The extent to which cancer patients trust in cancer-related online information: a systematic review
Source: PeerJ. 2019 Sep 30;7:e7634. doi: 10.7717/peerj.7634 (PMC6776066; doi:10.7717/peerj.7634)
Supplement: Table S3 [file peerj-07-7634-s003.docx]

**Supplemental Table S3:**

**Inclusion and exclusion criteria for full text screening.**

| Inclusion criteria | Number (n) of articles that are not matching the criteria |
| --- | --- |
| Full text accessible | 2 |
| The used language is German, English or French | 1 |
| The Article includes some quantitative data/ not just qualitative research | 4 |
| Participants are ≥ 18 years | 2 |
| A part of the participants are cancer patients with any type of cancer | 16 |
| If not all participants are cancer patients, then it is possible to distinguish between results of cancer patients and others | 4 |
| Measured construct is any form of trust | 20 |
| Measured construct is trust in online information/ internet information/ online health information etc. | 2 |
