# Supplementary material for: The extent to which cancer patients trust in cancer-related online information: a systematic review
Source: PeerJ. 2019 Sep 30;7:e7634. doi: 10.7717/peerj.7634 (PMC6776066; doi:10.7717/peerj.7634)
Supplement: Table S4 [file peerj-07-7634-s004.docx]

**Supplemental Table S4:**

**Reasons for excluding RTI item bank questions from the quality assessment of the studies.**

| Question No. | Question | Reason for exclusion |
| --- | --- | --- |
| 4 | Does the study fail to account for important variations in the execution of the study from the proposed protocol? | Not applicable: the included studies had no protocols |
| 5 | Was the outcome assessor not blinded to the intervention or exposure status of participants? | Not applicable: the included studies had only one arm and so do not include comparison groups |
| 7 | Was the length of follow-up different across study groups? | Not applicable: Cross sectional studies do not include a follow-up |
| 8 | In cases of high loss to follow-up (or differential loss to follow-up), was the impact assessed (e.g., through sensitivity analysis or other adjustment method) | Not applicable: none of the included studies had a follow-up |
| 10 | Are any important harms or adverse events that may be a consequence of the intervention/exposure missing from the results? | Not applicable: intervention or unintended exposure were not described in the included studies |
| 12 | Any attempt to balance the allocation between the groups or match groups (e.g., through stratification, matching, propensity scores). | Not applicable: the included studies had only one arm and so do not include comparison groups |
