# Supplementary material for: The extent to which cancer patients trust in cancer-related online information: a systematic review
Source: PeerJ. 2019 Sep 30;7:e7634. doi: 10.7717/peerj.7634 (PMC6776066; doi:10.7717/peerj.7634)
Supplement: File S1 [file peerj-07-7634-s005.pdf]

Do cancer patients trust online health information, or the internet as a source of information? A systematic review

Lukas Lange, Mona Peikert, Christiane Bleich, Holger Schult

Citation

Lukas Lange, Mona Peikert, Christiane Bleich, Holger Schult. Do cancer patients trust online health information, or the internet as a source of information? A systematic review. PROSPERO 2017 CRD42017070190 Available from:
http://www.crd.york.ac.uk/PROSPERO/display_record.php?ID=CRD42017070190

Review question

Do cancer patients trust online health/cancer information?
Do cancer patients trust online health/cancer information sources?
Does trust in online health information differ across patients of different ages, genders, educational levels or cancer diagnoses?

Searches

The following electronic databases will be searched, using pre-specified search terms without restrictions to the publication period: MEDLINE, CINAHL, Web of Science Core Collection, PsycINFO and PsycIndex.
Studies published in English, German, French or Dutch will be included in the review, and studies written in languages other than those mentioned above will be included if the abstract is written in English, German, French or Dutch, and if it is possible to extract the minimum information required for the review.
In addition, the reference lists of included papers will be hand searched for further relevant literature.

Types of study to be included

Studies will only be included if they measure cancer patients' self-reported trust in online health information, but studies reporting quantitative or mixed data will also be considered. Study designs to be included are: observational studies, randomised and non-randomised field and laboratory experiments, pilot studies, systematic reviews and meta-analyses.Inclusion: full text available, published in a peer reviewed journal, and written in German, French, Dutch or English.

Condition or domain being studied

Health websites are a popular destination for cancer patients searching for health-related information.
Recent research, however, suggests that a lack of trust in Internet information may be one reason why older adults underuse this resource (Donohue, Huskamp, Wilson, & Weissman, 2009; Hesse et al., 2005; Zulman, Kirch, Zheng, & An, 2011).
This review will focus on patient-reported measures of trust in online health information concerning their type of cancer.
Prior research has found that the sociodemographic factors of the consumer (age, income. education), their health status, website-related antecedents and consumer-to-website interaction-related antecedents will influence the extent of patients' trust in online health information (Kim, 2014).

Participants/population

Inclusion: adult cancer patients (>= 18 years) with any type of cancer, who have used the Internet at least once.
Exclusion: trust reported by health care professionals, patient's relatives and participants who do not have a cancer diagnosis will be excluded.

Intervention(s), exposure(s)

Not applicable.

Comparator(s)/control
Not applicable.

Context

Primary outcome(s)
Patients self-reported trust (mistrust, active trust) in online health information, and patients' self-reported trust in online health information sources.

Secondary outcome(s)
Not applicable.

Data extraction (selection and coding)
The titles and/or abstracts of relevant studies will be retrieved using the search strategy, and duplicates removed prior to data selection and extraction. A PRISMA flow diagram will be used to state the number of arcticles found, and those included and excluded, as well as the reasons for exclusion (at the full text screening stage), and EndNote will be used to keep track of all studies.
Selection: first, all titles and abstracts of the retrieved articles will be screened to identify studies which potentially meet the inclusion criteria. In the second step, two reviewers will independently assess the full texts of all potentially relevant articles. As stated above, the reasons for any exclusions, as well as the number of articles excluded, will be recoreded and presented in the PRISMA flow diagram. Disagreements between the two reviewers over the eligibility of studies will be resolved through discussion.
Extraction: the following data will be extracted from the eligible articles: 1. authors; 2. year of publication; 3. language; 4. study design; 5. sample size; 6. recruitment; 7. the questionnaire used; 8. the socio-demographics (age, gender, education, type of cancer and users and non-users) of the participants; 9. the medical characteristics (cancer entity, treatment intention, treatment etc.) of the participants; 10. consumer-to-website interaction-related antecedents; and 11. outcomes and results. Missing data will be requested from the athors of the relevant study, and if the missing data (especially concerning the outcomes) cannot be found, then the study will be excluded from the analysis.

Risk of bias (quality) assessment
The quality of the included studies will be assessed by two independent reviewers, using the RTI item bank, to evaluate the risk of bias, and the level of precision for the observational studies. Experimental studies will be assessed using the Cochrane risk of bias tool.
Disagreements between the reviewers over the assessment of the studies will be resolved by discussion.

Strategy for data synthesis
Descriptive information from all of the included studies will be presented in a table. The findings from the individual studies will be combined, and summarized using a narrative approach (Dixon-Woods, Agarwal, Jones, Young & Alex Sutton, 2005). A meta-analysis will be considered if it is possible to calculate the average trust in online information in a large enough proportion of the studies, and to standarize this information in a manner that will allow it to be synthesized across studies.

Analysis of subgroups or subsets
If the necessary data are available, subgroup analyses will be conducted for patients with different types of cancer, educational level, age, and health literacy.

Contact details for further information
Lukas Lange
lu.lange@uke.de

Organisational affiliation of the review
University Medical Center Hamburg-Eppendorf
https://www.uke.de/english/index.html

Review team members and their organisational affiliations

Mr Lukas Lange. University Medical Center Hamburg-Eppendorf
Ms Mona Peikert. University Medical Center Hamburg-Eppendorf
Dr Christiane Bleich. University Medical Center Hamburg-Eppendorf
Professor Holger Schult. University Medical Center Hamburg-Eppendorf

Anticipated or actual start date

15 December 2016

Anticipated completion date

31 August 2017

Funding sources/sponsors

The review is not funded by any organisation.

Conflicts of interest

None known

Language

English

Country

Germany

Stage of review

Review_Ongoing

Subject index terms status

Subject indexing assigned by CRD

Subject index terms

Consumer Health Information; Health Communication; Health Knowledge, Attitudes, Practice; Humans; Internet; Neoplasms; Patient Medication Knowledge; Telemedicine; Trust

Date of registration in PROSPERO

22 June 2017

Date of publication of this version

22 June 2017

Details of any existing review of the same topic by the same authors

Stage of review at time of this submission

Stage	Started	Completed
Preliminary searches	Yes	Yes
Piloting of the study selection process	Yes	Yes
Formal screening of search results against eligibility criteria	Yes	Yes
Data extraction	Yes	No
Risk of bias (quality) assessment	No	No
Data analysis	No	No

Versions

22 June 2017

PROSPERO
This information has been provided by the named contact for this review. CRD has accepted this information in good faith and registered the review in PROSPERO. CRD bears no responsibility or liability for the content of this registration record, any associated files or external websites.
