# Supplementary material for: The extent to which cancer patients trust in cancer-related online information: a systematic review
Source: PeerJ. 2019 Sep 30;7:e7634. doi: 10.7717/peerj.7634 (PMC6776066; doi:10.7717/peerj.7634)
Supplement: Supplemental Information 1 [file peerj-07-7634-s006.docx]

# Rationale for conducting the meata-analysis.

The Internet provides cancer patients with the information they may need about diagnosis, prognosis or likelihood of cure, disease stage and treatment options, or the side effects of treatment (Castleton et al. 2011; Maddock et al. 2011; Tariman et al. 2014). They have the possibility to acquire knowledge about their own health and develop the ability to influence, strengthen and actively participate in its treatment (empowerment) (Aujoulat et al. 2007; Lange et al. 2018). As the quality of the cancer-related information online is highly variable (Al-Bahrani & Plusa 2004; Borgmann et al. 2016; Bruce et al. 2015; Liebl et al. 2015; Wasserman et al. 2014), patients decisions regarding whether to act upon the information they find strongly depend on the trustworthiness of the information and its source (Lemire et al. 2008).

The systematic review that we conducted has an explorative character. The analysis of patients’ trust in cancer-related online information gives us the opportunity to describe the quality of cancer-related online information from a consumer perspective.

# The contribution that the meta-analysis makes to knowledge in light of previously published related reports, including other meta-analyses and systematic reviews.

To the best of our knowledge this is the first systematic review that analyzed cancer patients’ trust in cancer-related online information or in the internet as a source of cancer information. Our findings suggest that approximately half of cancer patients seem to trust information found on the internet or trust the internet itself as a source of cancer information. The content of cancer-related information online and the providers of this information are constantly changing. An analysis of the information quality by experts is always only a snapshot, due to the constant adaptation of content and providers. While the number of studies included in this review is relatively small (n=7), our synthesis gives a first impression on how the varying quality of cancer-related online information (Al-Bahrani & Plusa 2004; Borgmann et al. 2016; Bruce et al. 2015; Liebl et al. 2015; Wasserman et al. 2014) is perceived by the consumer.

# References

Al-Bahrani A, and Plusa S. 2004. The quality of patient-orientated internet information on colorectal cancer. *Colorectal disease : the official journal of the Association of Coloproctology of Great Britain and Ireland* 6:323-326. DOI: 10.1111/j.1463-1318.2004.00604.x.

Aujoulat I, d'Hoore W, and Deccache A. 2007. Patient empowerment in theory and practice: polysemy or cacophony? *Patient education and counseling* 66:13-20. DOI: 10.1016/j.pec.2006.09.008.

Borgmann H, Mager R, Salem J, Brundl J, Kunath F, Thomas C, Haferkamp A, and Tsaur I. 2016. Robotic prostatectomy on the web: A cross-sectional qualitative assessment. *Clinical genitourinary cancer* 14:e355-362. DOI: 10.1016/j.clgc.2015.12.020.

Bruce JG, Tucholka JL, Steffens NM, and Neuman HB. 2015. Quality of online information to support patient decision-making in breast cancer surgery. *Journal of surgical oncology* 112:575-580. DOI: 10.1002/jso.24046.

Castleton K, Fong T, Wang-Gillam A, Waqar MA, Jeffe DB, Kehlenbrink L, Gao F, and Govindan R. 2011. A survey of Internet utilization among patients with cancer. *Supportive care in cancer : official journal of the Multinational Association of Supportive Care in Cancer* 19:1183-1190. DOI: 10.1007/s00520-010-0935-5.

Lange L, Schulz H, and Bleich C. 2018. E-Health-Angebote in der Onkologie. *Der Onkologe*. DOI: 10.1007/s00761-018-0348-5.

Lemire M, Pare G, Sicotte C, and Harvey C. 2008. Determinants of Internet use as a preferred source of information on personal health. *International journal of medical informatics* 77:723-734. DOI: 10.1016/j.ijmedinf.2008.03.002.

Liebl P, Seilacher E, Koester MJ, Stellamanns J, Zell J, and Hubner J. 2015. What cancer patients find in the internet: the visibility of evidence-based patient information - analysis of information on German websites. *Oncology research and treatment* 38:212-218. DOI: 10.1159/000381739.

Maddock C, Lewis I, Ahmad K, and Sullivan R. 2011. Online information needs of cancer patients and their organizations. *Ecancermedicalscience* 5:235. DOI: 10.3332/ecancer.2011.235.

Tariman JD, Doorenbos A, Schepp KG, Singhal S, and Berry DL. 2014. Information needs priorities in patients diagnosed with cancer: a systematic review. *Journal of the advanced practitioner in oncology* 2014:115-122.

Wasserman M, Baxter NN, Rosen B, Burnstein M, and Halverson AL. 2014. Systematic review of internet patient information on colorectal cancer surgery. *Diseases of the colon and rectum* 57:64-69. DOI: 10.1097/DCR.0000000000000011.
